# Cytotoxic innate intraepithelial lymphocytes control early stages of *Cryptosporidium* infection

**DOI:** 10.3389/fimmu.2023.1229406

**Published:** 2023-09-06

**Authors:** Fatima Hariss, Marie Delbeke, Karine Guyot, Pauline Zarnitzky, Mohamad Ezzedine, Gabriela Certad, Bertrand Meresse

**Affiliations:** ^1^ Univ. Lille, Inserm, CHU Lille, U1286 - INFINITE - Institute for Translational Research in Inflammation, Lille, France; ^2^ Institut Pasteur de Lille, U1019-UMR 9017-CIIL-Centre d’Infection et d’Immunité de Lille, University of Lille, Lille, France; ^3^ Department of Biology, Faculty of Science, Lebanese University, Beirut, Lebanon; ^4^ Délégation à la Recherche Clinique et à l’Innovation, Groupement des Hôpitaux de l’Institut Catholique de Lille, Lomme, France

**Keywords:** gut, innate intraepithelial lymphocytes, cryptosporidium, organoids, cytotoxicity

## Abstract

**Background:**

Intraepithelial lymphocytes (IELs) are the first immune cells to contact and fight intestinal pathogens such as *Cryptosporidium*, a widespread parasite which infects the gut epithelium. IFN-γ producing CD4^+^ T IELs provide an efficient and a long-term protection against cryptosporidiosis while intraepithelial type 1 innate lymphoid cells limits pathogen spreading during early stages of infection in immunodeficient individuals. Yet, the role of T-cell like innate IELs, the most frequent subset of innate lymphocytes in the gut, remains unknown.

**Methods:**

To better define functions of innate IELs in cryptosporidiosis, we developed a co-culture model with innate IELs isolated from *Rag2^-/-^* mice and 3D intestinal organoids infected with *C. parvum* using microinjection.

**Results:**

Thanks to this original model, we demonstrated that innate IELs control parasite proliferation. We further showed that although innate IELs secrete IFN-γ in response to *C. parvum*, the cytokine was not sufficient to inhibit parasite proliferation at early stages of the infection. The rapid protective effect of innate IELs was in fact mediated by a cytotoxic, granzyme-dependent mechanism. Moreover, transcriptomic analysis of the Cryptosporidium-infected organoids revealed that epithelial cells down regulated Serpinb9b, a granzyme inhibitor, which may increase their sensitivity to cytolytic attack by innate IELs.

**Conclusion:**

Based on these data we conclude that innate IELs, most likely T-cell-like innate IELs, provide a rapid protection against *C. parvum* infection through a perforin/granzymes-dependent mechanism. *C. parvum* infection. The infection may also increase the sensitivity of intestinal epithelial cells to the innate IEL-mediated cytotoxic attack by decreasing the expression of Serpin genes.

## 1 Introduction

Intestinal intraepithelial lymphocytes (IELs) are tissue resident memory cells which localize within the epithelial layer all along the digestive tract. Owing to their strategic position, their effector and regulatory functions, IELs are considered as the guardians of the gut. Notably, IELs play a potent role in host defense as they can respond rapidly and efficiently to a large variety of pathogens such as viruses, bacteria, fungi, and parasites (1, 2). This property certainly relies on the heterogeneity of the IEL population which is mainly formed by two T cell subsets named conventional (_C_IEL) and nonconventional (_NC_IEL) IELs. Conventional IELs are similar to the effector/memory TCRαβ+ cells from the other compartments and are stimulated by microbial peptides presented by MHC. In contrast, in mice, _NC_IELs express the homodimer CD8αα with either a TCRαβ or a TCRγδ. Most of them recognize self-antigens or proteins from pathogens independently of a classical MHC (1, 2). The IEL compartment also contains lymphoid cells which do not express a TCR. This sub-population of IELs is mainly composed of type 1 innate lymphoid cells (ILC1) expressing the natural cytotoxicity receptor NKp46 and of peculiar innate lymphocytes with T cell features, named T-cell-like innate IELs (3, 4). The latter population is dominant and expresses intracellular CD3γ and the integrin CD103 (α_E_β7). Around half of them are CD8αα^+^ (iCD8α) (4, 5). While ILC1s express high level of IFN-γ, T-cell-like IELs produce granzymes and are cytotoxic (4). ICD8α also have the capacity to produce osteopontin encoded by *Spp1* which sustains the homeostasis of ILC1 (6), to phagocyte bacteria and to process and present antigens to MHC class II-restricted T cells (5). Yet, the role of T-cell-like innate IELs in infection remains poorly studied.

*Cryptosporidium* is an apicomplexan parasite and an opportunistic pathogen that infects the gut epithelium. It is recognized as one of the most important waterborne contaminants in the world and a major cause of diarrhea in human and animals. Since *Cryptosporidium* infects enterocytes by their apical side and replicate within the epithelium, IELs are crucial to detect and fight the parasite (7–9). *Cryptosporidium* specific CD4+ T _C_IELs are able to eliminate the parasite by secreting IFN-γ and thus provide an efficient and a long-term protection (9). When the adaptive immune response is impaired the infection is chronic and much more severe (10–13). However, in immunodeficient individuals, innate mechanisms limit the replication of the parasite. Notably, a number of studies have pinpointed the protective roles of intestinal epithelial cells (iEC), mononuclear phagocytes, neutrophils and conventional Natural Killer (cNK) cells (14, 15). Yet, there are conflicting data about the contribution of cNK cells in cryptosporidiosis since the depletion of cNK cells using anti-asialoGM1 antibodies in immunodeficient SCID or *Rag2^-/-^* mice does not impact the course of the infection (16–18). Actually, ILC1s but not cNK cells seem to protect against *Cryptosporidium*. Indeed, a recent work showed that ILC1s limit the expansion of the parasite in *Rag2^-/-^* animals through their secretion of IFN-γ (18).Yet, the role of innate IELs in cryptosporidiosis remains poorly studied. Herein, we developed an *in vitro* model to specifically investigate their functions during the infection. The model is based on the co-culture of innate IELs isolated from *Rag2^-/-^* mice with murine 3D intestinal organoids infected with *Cryptosporidium parvum*. Using this original experimental assay, we showed that innate IELs rapidly prevent the expansion of the parasite. Interestingly, the protection mediated by IFN-γ produced by ILC1s was not essential during the very early stage of the infection. Instead, we found that the protective effect mostly depends on perforin and serine proteases such as granzymes. Moreover, we also found that infected iEC down regulate the natural granzyme inhibitor serpinb9b and thus could be more sensitive to IELs mediated cytotoxicity.

## 2 Material and methods

### 2.1 Mice

Females *Rag2^-/-^* C57BL/6 and C57BL/6 WT mice were obtained from a colony bred at the Pasteur Institute of Lille (France) and regularly controlled for microbial or parasitological pathogens. Animals were housed in groups in covered cages and maintained under aseptic conditions with standard laboratory food and water. The animal experiment ethics committee approved the experimental animal study protocol (APAFIS#30539).

### 2.2 *In vivo* infection of *Rag2^-/-^* C57BL/6 mice

Eight-week-old *Rag2^-/-^* mice were infected by oral gavage with 5x10^4^ *C. parvum* oocysts (Iowa strain) per mouse (n=15 infected and n=14 controls). Twenty-four hours post infection (PI), mice were euthanized and the small intestine from each mouse was collected. Ileal sections were collected to quantify the number of innate immune cells in the epithelium by immunohistochemistry and to quantify the parasitic load and the expression of cytokines by RT-qPCR. Innate IELs were isolated as described below and used to quantify gene expression by RT-qPCR and to define their phenotype by flow cytometry.

### 2.3 Isolation of intestinal crypts and culture of intestinal organoids

Intestinal crypts were isolated from small intestine of female C57BL/6 mice as described by Sato et al. (19). Briefly, small intestine fragments were incubated with PBS 1X containing 8mM of EDTA and shaked for 1h on ice using a rocking platform. Then, EDTA buffer was removed, tissue fragments were vigorously resuspended in cold PBS 1X and supernatant was collected to quantify the number of crypts. One thousand crypts were cultured in 30μl of Matrigel (Corning). The Matrigel was polymerized for 10 minutes at 37°C, and 600µl/well of LWRN conditioned medium was added. The Rho-associated kinase inhibitor Y-27632 (10 μmol/L; Tocris) was included in the medium for the first 2 days to avoid anoikis.

Organoids were passed once a week by dissociating the Matrigel for five minutes at 37°C with TrypLE Express (Gibco, Life Technologies).

### 2.4 Microinjection of intestinal organoids with *C. parvum* oocysts

*C. parvum* IOWA oocysts were purchased from WaterborneTM, Inc. (New Orleans, Louisiana). Oocyst solution was stored in the shipping medium (phosphate buffered saline or PBS with penicillin, streptomycin, gentamycin, amphotericin B and 0.01% Tween 20) at 4°C until use. For microinjection, 250 oocysts/µl of the stock solution were centrifuged at 2000 g for 10 minutes. After treatment with 0.025% of Trypsin pH=2.4 (Sigma) for 20 minutes at 37°C, oocysts were resuspended with excystation medium containing RPMI 1640 with 2 mM of L-glutamine (Gibco), 1% of fetal calf serum, 100 mg/ml of penicillin/streptomycin (Gibco), 0.25 mg/ml of Gentamycin (Dutscher), 0.2mg/ml of Bovine Bile (Acros Organics), 1mg/ml of glucose (BioXtra), 0.25µg/ml of folic acid (Alfa Aesar), 1µg/ml of 4-aminobenzoic acid (VWR), 8.75µg/ml of L-Ascorbic acid (Sigma Aldrich) and 0.5µg/ml Calcium Pantothenate (Acros Organics) (20).

A sterile glass capillary of 15μm diameter was used for microinjection (Transfer tip eppendorf). The capillary was loaded with oocysts (250 oocysts/µl) suspended in their excystation medium containing 25 µg/ml of Fast green dye (Sigma) in order to visualize micro-injected organoids. Approximately 200 nl of suspension was injected into each organoid using the Leica DMI 4000B microinjector. For each experiment 20 to 30 organoids were cultured in IbiTreat microdish (Ibidi) and 50% of them were microinjected.

### 2.5 Isolation of innate IELs and co-culture with intestinal organoids

Isolation of murine innate IELs was performed according to the method described by Schulthess et al., 2012 (21). Briefly, small intestines of 8 weeks old *Rag2^-/-^* mice were removed and washed with cold PBS 1X. Mesenteric fat and Peyer’s patches were removed. The intestine was then opened longitudinally and cut into 0.5 cm fragments which were then incubated in 50 ml of RPMI (Gibco) containing 10% FCS for 2h at 37°C with vigorous agitation. The supernatant is passed through a glass wool column to remove part of iECs. Cells were then separated on a gradient 40/80% of Percoll (GE Healthcare). The innate IELs ring was then collected, washed and taken up in 1ml RPMI-10% FCS. Then 10^5^ innate IELs were co-cultured with infected or non-infected organoids 24 hours PI. The co-culture was stopped after 24h. To inhibit IFN-γ or cytolytic activities, anti-IFN-γ Ab (10 µg/ml) (clone XMG1.2 Biolegend) or granzyme B inhibitor I (10µM) (Merck) or aprotinin (2µg/ml) (Sigma) was added in the co-culture simultaneously with IELs. Concanamycin A treatment: innate IELs were isolated from *Rag*2^-/-^ mice and treated for 3h with 50nM of concanamycin A (CMA) (Biotechne) at 37°C. Treated-innate IELs were then washed 2 times and co-cultured with infected organoids for 24h.

### 2.6 RNA isolation and RT–qPCR

Total RNA was extracted from organoids using a Nucleospin^®^RNA II kit (Macherey-Nagel) according to manufacturer’s protocol. Complementary DNA was synthesized from 1 μg total RNA using a High Capacity cDNA Reverse Transcription Kit (Applied Biosystems). Real time PCR was performed using Power SYBR Green PCR Master Mix in a StepOne plus system (Applied Biosystems). Gene expression was quantified using the ΔΔ Ct method for rRNA 18s. Cp18S forward, 5’- TGCCTTGAATACTCCAGCATGG-3’; Cp18S reverse, 5’-TACAAATGCCCCCAACTGTCC-3’. The expression of other genes was quantified using ΔCt method. The gene coding for murine beta-actin (*β-actin*) was used as housekeeping gene (**Table 1**).

**Table 1:** Forward and reverse primer sequences for RT-qPCR.

Gene	Primer sequences
*Actb*	F: CCTTCTTGGGTATGGAATCCT R: CTTTACGGATGTCAACGTCAC
*Ifng*	F: ATGAACGCTACACACTGCATC R: CCATCCTTTTGCCAGTTCCTC
*Spp1*	F: TCTGATGAGACCGTCACTGC R: AGGTCCTCATCTGTGGCATC
*Serpinb9b*	F: GATGATTGCCAGCTAGATTG R: TGACCACATAATGTCTGGTTTG
*Ifna*	F: GTGCTGGCTGTGAGGACA R:GGCTCTCCAGACTTCTGCTCT
*Gzmb*	F: CAGCAAGTCATCCCTATGGT R: TACTCTTCAGCTTTAGCAGCAT
*Cd8a*	F: TTTACATCTGGGCACCCTTG R: CTTTCGGCTCCTGTGGTAG
*Itgae*	F: GACAAAGACTCAGGACCACAC R: GGCCACGGTTACATTTTCTTT
*Ncr1*	F: GATCAACACTGAAAAGGAGACT R: TGACACCAGATGTTCACCGA

### 2.7 Confocal microscopy

Innate IELs isolated from the *Rag2^-/-^* mice were labeled with 5 μM CellTrace CFSE (Invitrogen) for 20 min at 37° C. One hundred thousand cells were co-cultured with organoids. After 24 h of culture, organoids were fixed with 4% paraformaldehyde (Microm microtech) for 30 min at RT. Organoids were then permeabilized with PBS 1X containing 1% of triton 100X (Sigma) for 10 min at RT. After washing, organoids were labeled with DAPI (Thermo Fisher) and 1.65 μM of phalloidin Alexa Fluo 647 (Invitrogen) for 1 hour at RT. The co-culture was visualized under a Leica Sp8 confocal microscope.

### 2.8 Flow cytometry and Cell-sorting

Cells were first incubated with anti-mouse CD16/CD32 Ab (clone 2.4 G2, BD Biosciences) for 10 min at 4° C, then washed and labeled with a cocktail of antibodies for 20 min at 4°C in dark (see **Table 2**). Cells were washed and treated with BD FACS Lysing Solution (BD Biosciences) for 5 minutes at RT. After washing, cells were analyzed on the LSR Fortessa X20 cytometer (Becton Dickinson).

**Table 2:** Antibodies used for flow cytometry.

Manufacturer	Cat#	Antibodies	Clone
Sony	1115540	FITC anti-mouse CD45	30-F11
Biolegend	100713	APC/Cyanine anti-mouse CD8a	53-6.7
Sony	1207125	Pe/Cy7 anti-mouse CD103	2E7
Biolegend	137611	Brilliant Violet 421 anti-mouse CD335 (NKp46)	29A1.4

For cell sorting, innate IELs were labeled with a cocktail of antibodies (CD45 FITC (Sony), CD103 PeCy7 (Biolegend) and NKp46 BV421 (Biolegend). Four populations (CD45^+^, CD45^+^NKp46^-^, CD45^+^CD103^-^NKp46^-^, and CD45^+^CD103^+^) were sorted using a BD FACSAria II SORP cell sorter (Becton Dickinson).

### 2.9 Immunohistochemistry

Ileal samples from all mice of each group were fixed with 4% paraformaldehyde and then embedded in paraffin. Four micrometer-thick sections were incubated with citrate 1X antigen repair solution (Skytec) at 95°C for 20 min after dewaxing and hydration. Then endogenous peroxidase was blocked by Bloxall blocking solution (Vector) for 10 min at RT. Nonspecific antigens were blocked with 5% goat serum for 30 min. The sections were exposed to primary anti-CD3γ (Abcam) and anti -CD8α (Cell Signaling Technology) Abs overnight at 4°C. After washing with Tris-buffered saline solution containing 0.05% Tween, sections were incubated for 30 min with the detection kit “Immpress peroxidase Polymer anti rabbit IgG” (Vector). Negative controls were incubated with irrelevant serum. The staining was revealed using the peroxidase substrate, DAB (Cell Signaling Technology). Hematoxylin counterstain was performed before mounting the slides in an aqueous medium. Slides were analyzed using a microscopy (Leica).

### 2.10 Quantification of IFN-γ

The secretion of IFN-γ was measured in supernatants of co-cultures using “Mouse IFNγ ELISA MAXTM Deluxe set” kit (Biolegend).

### 2.11 LDH release assay

Cell death was determined using Cytotox96 non-radioactive cytotoxicity assay (Promega) following the manufacturer’s protocol. The colorimetric assay quantified lactate dehydrogenase (LDH) activity released from the cytosol of damaged target cells into the supernatants. Briefly, after 48h of infection, 50µl of co-culture supernatant were incubated with 50 µl of CytoTox 96 reagent for 30 min. The reaction was stopped and the absorbance was recorded at 490nm on Fluostar Omega spectrophotometer (BMG Labtech).

### 2.12 RNA sequencing

Starting from 4µl of total RNA we add 1µl of ERCC spike-in control. Library generation is then initiated by oligo dT priming, from total RNA (between 50 and 200 ng). The primer already contains Illumina- compatible linker sequences (Read 2). After first strand synthesis the RNA is degraded and second strand synthesis is initiated by random priming and a DNA polymerase. The random primer also contains 5’ Illumina-compatible linker sequences (Read 1). At this step Unique Molecular Identifiers (UMIs) are introduced allowing the elimination of PCR duplicates during the analysis. After obtaining the double stranded cDNA library, the library is purified with magnetics beads and amplified. During the library amplification the barcodes and sequences required for cluster generation (index i7 in 3’ and index i5 in 5’) are introduced due to Illumina- compatible linker sequences. The number of cycles depends on the starting quantity, between 14 cycles for 200ng of total RNA and 16 cycle for 50ng of total RNA. If the quantity is less than 50 ng, the number of cycles will be increase (for example for 17ng, 17 cycles). The final library is purified and deposed on High sensitivity DNA chip to be controlled on Agilent bioanalyzer 2100. The library concentration and the size distribution are checked.

Each library is pooled equimolarly and the final pool is also controlled on Agilent bioanalyzer 2100 and sequenced on NovaSeq 6000 (Illumina) with 100 cycles chemistry. Different chips can be used for sequencing, it depends on the number of libraries pooled, the objective is to obtain a minimum of 20 M reads by sample.

To eliminate poor quality regions and poly(A) of the reads, we used the fastp program. We used quality score threshold of 20 and removed the read shorter than 25 pb. The read alignments were performed using the STAR program with the genome reference mouse (GRCm39) and the reference gene annotations (Ensembl). The UMI (Unique Molecular Index) allowed to reduce errors and quantitative PCR bias using fastp and umi-tools. Based on reads alignments, we counted the numbers of molecules by gene using FeatureCount. Other programs were performed for the quality control of reads and for the workflow as qualimap, fastp, FastQC and MultiQC. Differential Gene Expression of RNA-seq was performed with R/Bioconductor package DESeq2. The cut-off for differentially expressed gene was p-value padj (BH) < 0.1. RNA sequencing data that support the findings of this study have been deposited in sequence Read Archive (SRA) data (https://dataview.ncbi.nlm.nih.gov/object) with the accession code PRJNA98061.

### 2.12 Statistical analysis

Data were expressed as the mean ± SD or the median with range. A Wilcoxon matched-pairs signed rank test was used for *in vitro* experiments. Statistical analyses were performed using StatXact software (Cytel Studio 7) and GraphPad Prism software (version 5.0, San Diego, CA, USA). The threshold for statistical significance was set to p<0.05.

## 3 Results

### 3.1 IFN-γ, osteopontin and granzyme B expressions are up regulated at early stages of *Cryptosporidium* infection in *Rag2^-/-^* mice

**Figure 1:**
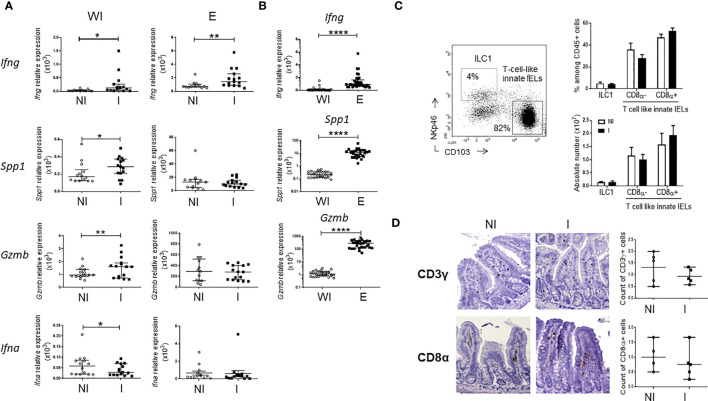
Very early immune responses induced by *C. parvum* infection in the ileum of *Rag2^-/-^* mice. *Rag2^-/-^* mice were infected by oral gavage with *C. parvum* for 24h. Quantitative RT-qPCR analysis was performed to compare expression of genes in the whole small intestine (WI) and in the epithelium (E) **(A)** between non-infected (NI) (n=14) and infected (I) (n=15) mice and **(B)** between sites. Results were pooled from 3 independent experiments. Medians and ranges are shown. **(C)** Frequencies of innate IELs subsets in the small intestine of NI (n=4) and I (n=5) Rag2^-/-^mice using flow cytometry; representative dot plots and histograms of means values. **(D)** Immunohistochemistry, staining of CD3γ and CD8α on ileal sections from NI (n=5) and I (n=5) *Rag2^-/-^* mice. Scatter plots summarizes results and average values. ****p < 0.00005, **p < 0.005 and *p < 0.05.

To identify immune mechanisms activated during the very early phase of *C. parvum* infection, we first analyzed the expression of genes related to key effector functions of innate immune cells such as interferons and granzymes in the small intestine of adult *Rag2^-/-^* mice 24h post-infection (PI). We found that expression of IFN-γ (*Ifng*) and Osteopontin (*Spp1*), two molecules associated with the type 1 immune response, as well as the cytolytic enzyme granzyme B (*Gzmb*) were rapidly increased in the ileum of infected animals. In contrast, the amount of IFN-α mRNA which is mainly produced by enterocytes and DC in infected mice (22) was decreased (**Figure 1A**). Yet, only the expression of IFN-γ was significantly increased in cells isolated from the epithelium of *Rag2^-/-^* mice infected with *Cryptosporidium* (**Figure 1A**). However, levels of *Spp1* and *Gzmb* mRNA were around 50 and 200 times higher in the epithelium than in the whole intestine respectively, indicating that cells expressing these genes are enriched (**Figure 1B**).

In the intestinal epithelium, ILC1s secrete high amounts of IFN-γ while T-cell-like innate IELs, including iCD8a, are known to produce osteopontin and granzyme B (23). We then studied whether NKp46^+^CD103^-^ ILC1s (3) and innate CD103^+^CD8α^+/-^ IELs (4, 5) expand in the epithelium upon infection. Frequencies and absolute numbers of these subsets were identical in the gut epithelium of infected and non-infected *Rag2^-/-^* mice (**Figures 1C, D**; **Supplementary Figures 1, 2**).

Together, these data show that type 1 and cytolytic immune responses are activated in few hours after *C. parvum* infection.

### 3.2 Innate IELs control *C. parvum* infection in co-culture with intestinal organoids

**Figure 2:**
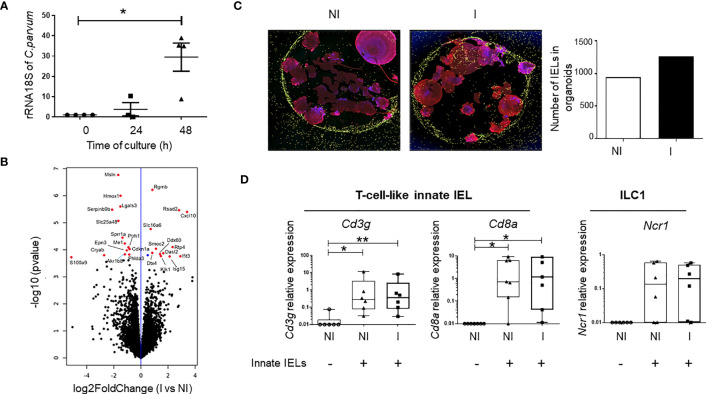
Innate IELs controls *C. parvum* infection in murine intestinal organoids. **(A)** The parasitic load was measured at 0, 24- and 48-hours PI in intestinal organoids using RT-qPCR to quantify *C. parvum* 18S rRNA. Results were normalized to the β-actin transcript level and median with range are shown in 4 independent experiments. **(B)** Volcano plot of differentially expressed genes (DEGs) between non-infected (n=4) and infected (n=4) organoids. (blue dot padj ≤ 0.1; red dots FC ≥ 1.5 and padj ≤ 0.1). **(C)** Immunofluorescence of co-cultures with non-infected (NI) and organoids infected (I) for 24h with *C. parvum*. Nuclei are stained in blue (DAPI), the actin in red (phalloidin) and innate IELs in yellow (CFSE). The histogram shows the number of innate IELs present in organoids. **(D)** Expression of innate IELs signature genes (*Cd3g*, *Cd8a* and *Ncr1*) in co-cultures (n=6) with NI and I organoids using RT-qPCR. **p < 0.005 and *p < 0.05).

To demonstrate the protective role of innate IELs during the first stages of *C. parvum* infection, we developed an *in vitro* model in which small intestinal organoids infected with the parasite were co-cultured with innate IELs isolated from naïve *Rag2^-/-^* mice. Oocysts and sporozoites were microinjected inside the lumen of murine organoids in order to access to the apical side of iEC (**Supplementary Figure 3**, **Supplementary Movie 1**). The parasitic load increased gradually and was significantly up-regulated 2 days after the microinjection demonstrating that *C. parvum* infects and replicates within murine intestinal organoids (**Figure 2A**). Moreover, analysis of organoids’ transcriptomes using 3’RNA sequencing (RNA-seq) revealed that *C. parvum* infection modified significantly the expression of 26 genes (**Figure 2B**; **Supplementary Figure 4**). Most of them were involved in immune responses such as C-X-C motif ligand 10 (*Cxcl10*) gene that promotes the recruitment of immune cells (**Figure 2B**; **Supplementary Table 1**). Yet, the number of innate IELs was not significantly increased in infected organoids compared to non-infected ones. In fact, innate IELs migrates spontaneously and similarly into infected and non-infected organoids (**Figure 2C**; **Supplementary Figure 5**). Moreover, the expression of gene signatures of T-cell-like innate IELs (i.e. *Cd3g, Cd8a*) and of ILC1 (i.e. *Ncr1*) revealed that the two subsets were present in the same proportion within infected and non-infected organoids (**Figure 2D**).

**Figure 3:**
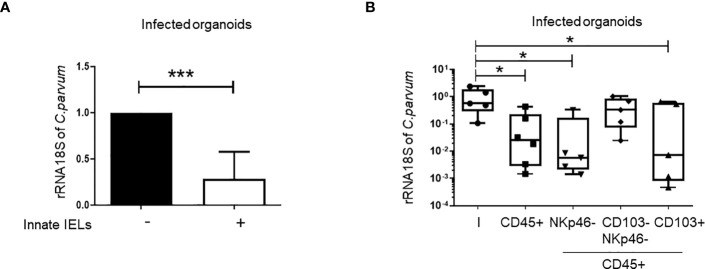
Innate IELs limit the development of *C parvum* in intestinal murine organoids. *C parvum* 18S rRNA expression was measured by RT-qPCR in infected organoids co-cultured or not with innate IELs (n=8). Results were normalized to the condition without lymphocytes and histograms present medians with interquartile range **(A)**. Innate IELs were FACS-sorted and co-cultured with infected organoids (n=2); Results are represented using box and whiskers show individual values **(B)**. *p < 0.05; ***p < 0.0005.

Importantly, the co-culture with innate IELs isolated from *Rag2^-/-^* mice decreased strikingly the amount of *C. parvum* in organoids (**Figures 3A, B**). To determine which subset protects against *C. parvum* infection, innate IELs from *Rag2^-/-^* mice were next sorted by FACS and co-cultured with infected organoids. As expected, purified CD45+ innate IELs significantly decreased the parasitic loads. This protective effect was not modified by the depletion of NKp46^+^ ILC1s but it was completely abolished after the removal of both ILC1s and CD103^+^ innate IELs. In addition, co-culture with purified CD103^+^ innate IELs, also tended to decrease the expansion of *C. parvum* within organoids (**Figure 3B**).

**Figure 4:**
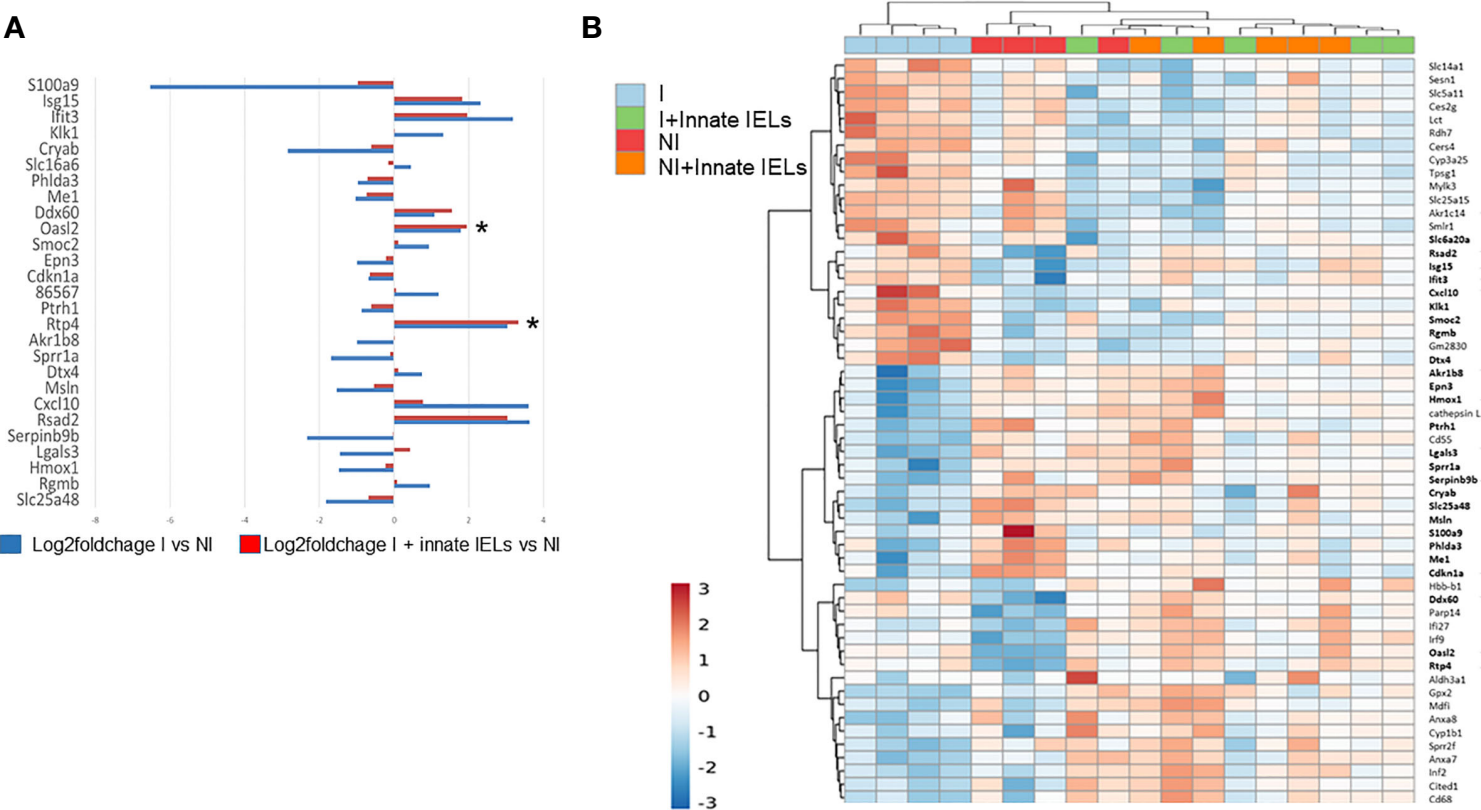
Innate IELs normalize expression of genes deregulated by *C parvum* in intestinal murine organoids. **(A)** Medians of log-fold changes of gene expression between infected organoids alone (in blue) or co-cultured with innate IELs (in red) and non-infected organoids. Twenty-seven genes differentially expressed between infected vs non-infected organoids are shown. **(B)** Heat map representing an unsupervised, hierarchical cluster analysis of all experimental conditions (p-value adjusted ≤ 0.1 and FC ≥ 1.5). *p < 0.05.

In keeping with the decrease of the parasitic load, the expression of genes which were modified by *C. parvum* infection in organoids was normalized by the presence of the innate IELs (**Figure 4A**). The unsupervised hierarchical clustering analysis also showed that transcriptomes of infected co-cultures were closer from that of non-infected organoids than from infected ones (**Figure 4B**).

Overall, these results showed that CD103^+^ innate IELs, the majority of which are T-cell-like innate IELs (4), protected against *C. parvum* infection in a co-culture model with intestinal organoids.

### 3.3 Co-culture revealed that the early protection against *C. parvum* infection does not depend on IFN-γ

**Figure 5:**
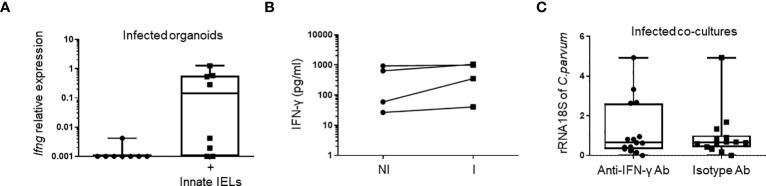
The anti-parasitic effect of innate IELs is independent of IFN-γ in co-cultures. Quantification of IFN-γ (*Ifng*) **(A)** using RT-qPCR (n=8) and **(B)** ELISA (n=4) in infected organoids with or without innate IELs. **(C)** Amounts of *C parvum* 18S rRNA in infected organoids co-cultured with innate IELs treated with a blocking anti-IFN-γ mAb or an isotype control Ab. Results show individual points from 3 independent experiments.

IFN-γ plays a key role in controlling of *Cryptosporidium* infection in both immunocompetent (9, 24, 25) and immunodeficient mice (16, 18). We also found a significant increase of *Ifng* expression in the gut of *Rag2^-/-^* 24h after the infection with *C. parvum* (**Figure 1A**), suggesting that the cytokine may also be involved in the early immune response against the parasite. Intraepithelial ILC1 produces high amount of IFN-γ and thereby limits parasite spreading (18). However, those cells were barely detectable in organoids (**Figure 2D**) and their depletion did not affect the protective effect of innate IELs in co-culture (**Figure 3B**). Nevertheless, CD103^+^ T-cell-like innate IELs were present in organoids (**Figure 2D**) and these cells can also produce IFN-γ, although in a smaller amount than ILC1s (6) (**Supplementary Figure 6**). Thus, we first seek the presence of IFN-γ in co-cultures. IFN-γ was detected in co-cultures with innate IELs but not in organoids alone (**Figure 5A**). The amount of IFN-γ released by innate IELs in the medium was however similar in co-cultures with organoids infected or not with *C. parvum* (**Figure 5B**). Moreover, blocking IFN-γ with a neutralizing Ab did not inhibit the protecting effect of innate IELs (**Figure 5C**).

Thus, very early protection mediated by innate IELs does not seem to rely on IFN-γ secretion.

### 3.4 Cytotoxic innate IELs provide rapid protection against *C. parvum*

**Figure 6:**
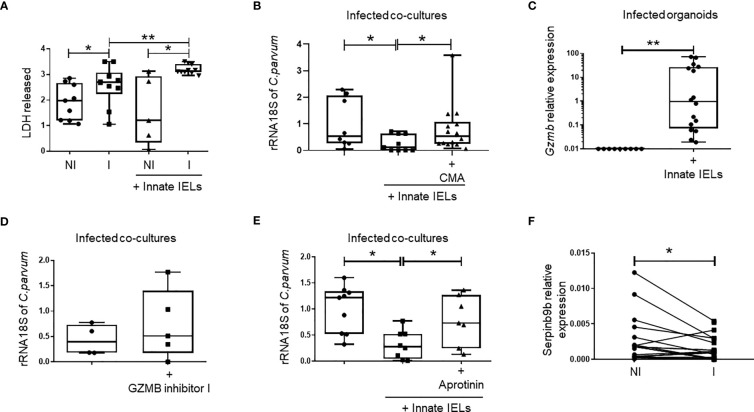
Innate IELs limit *C parvum* expansion in intestinal organoids through perforin and a serine-protease dependent mechanism. **(A)** Quantification of LDH release (DO 490 nm) after 48h of infection in supernatant of organoids non-infected (NI), infected **(I)** alone or co-cultured with innate IELs. (n=9). **(B)** Quantification of *C parvum* 18S rRNA using RT-qPCR in infected organoids co-cultured with innate IELs treated or not with the perforin inhibitor Concanamycin A (CMA) (n=2 independent experiments). **(C)** Expression of *Gzmb* in infected organoids cultured with or without innate IELs using RT-qPCR. Quantification of *C parvum* 18S rRNA using RT-qPCR in infected organoids co-cultured with innate IELs treated or not with **(D)** the GZMB inhibitor I or **(E)** with Aprotinin (n=3 independent experiments). Box and whiskers show individual value. **(F)** Expression of Serpinb9b measure by RT-qPCR in organoids infected or not for 24h with *C parvum* (n=10). **p < 0.005 and *p < 0.05.

T-cell-like innate IELs are cytotoxic cells (4, 5) and thus, they could reduce *Cryptosporidium* load by lysing infected iEC, alike cytotoxic NK (26, 27) and CD8 T cells (28, 29). In keeping with this hypothesis, we observed that cell death, measured as lactate dehydrogenase (LDH) release, was higher when innate IELs were co-cultured with infected organoids than when they were cultured with non-infected ones (**Figure 6A**). The level of LDH was also increased in organoids alone upon infection showing that *C. parvum* induces iEC death by itself. However, the quantity of LDH was significantly more elevated in infected organoids in presence of innate IELs indicating that the immune cells are cytotoxic and promote the exclusion of infected EC (**Figure 6A**).

To further analyze the cytotoxic mechanism, we next pre-treated innate IELs with the vacuolar type H+-ATPase inhibitor concanamycin A (CMA) before the co-culture with infected organoids. CMA inhibits cytotoxicity as it blocks perforin activity (30, 31). Since CMA-treated innate IELs were unable to control *C. parvum* infection (**Figure 6B**), we concluded that the immune response against the parasite likely relies on a perforin-dependent cytotoxic mechanism.

Alike perforin, granzyme B is a potent mediator of cytotoxicity in T-cell-like innate IELs (4, 5). Moreover, its expression was rapidly increased in the gut of *Rag2^-/-^* mice infected with *C. parvum* (**Figure 1A**) and granzyme B mRNA was detected in co-cultures within infected organoids (**Figure 6C**). To investigate the impact of granzyme B-mediated cytotoxicity on the infection, we then compared the parasitic load in co-cultures treated or not with the granzyme B specific inhibitor I. We found a small but not significant increase of *C. parvum* 18S rRNA in infected samples treated with the granzyme B inhibitor suggesting that other protective mechanisms are involved (**Figure 6D**).

Innate IELs also express additional cytolytic granzymes such as granzymes A (5). We then used aprotinin, a non-selective serine-protease inhibitor, to inhibit the activity of all granzymes expressed by innate IELs. Strikingly, aprotinin abolished the protective effect of innate IELs in co-culture indicating that they control the infection through a granzyme-dependent cytotoxic mechanism (**Figure 6E**).

Interestingly, RNA-seq showed a significant decreased of serpinb9b expression, a natural serine protease inhibitor, in intestinal organoids infected with *C. parvum* (**Figures 4A, B**). This result was further confirmed using RT-qPCR (**Figure 6F**). In addition, the infection seems to down regulate the expression of other serpins such as serpinb9 and b6b which inhibit the granzymes B and A, respectively (32) (**Supplementary Figure 7**).

Altogether, our data indicated that innate IELs protect against *Cryptosporidium* infection through a serine protease-dependent mechanism and suggest that infected iEC may be more sensitive to granzyme-mediated cytolysis.

## 4 Discussion

The intestinal epithelium contains many subsets of lymphocytes including adaptive conventional and unconventional T cells and innate IELs which maintains the homeostasis and ensure the protection of the compartment against a wide range of pathogens. Innate lymphocytes play a potent role in early stages of infection (16, 18) and they can also compensate for an immature or an impaired adaptive immunity. These properties are well shown in apicomplexan parasitosis in which NK and ILCs limit parasites spreading and expansion through the secretion of IFN-γ and cytotoxic mechanisms in WT and immunodeficient mice (33). Still, while intestinal Apicomplexa parasites (e.g. *Toxoplasma gondii*, *C. parvum*) infect and replicate in the gut epithelium, the role of innate IELs in these pathologies, which can be chronic and severe in immunocompromised individuals, remains poorly studied.

Investigating functions of intestinal innate IELs is challenging using *in vivo* experimental models since there is no efficient way to specifically deplete or modulate their activity. Moreover, the presence of cells with similar traits such as cNK, ILC3 and ILC1 in the lamina propria can hide their specific role. Thus, to dissect functions of innate IELs in cryptosporidiosis, we developed a co-culture system with murine small intestine organoids infected by *C. parvum*. We showed that *C. parvum* replicated within murine organoids like in human organoids (34) and stimulated immune mechanisms. Notably, the amount of *Cxcl10* mRNA, a chemokine usually induced by IFN-γ, was significantly increased in infected organoids indicating that iECs are a primary source of the chemokine and that the parasite directly stimulates its expression. This mechanism which has been reported by Lacroix-Lamandé et al. in murine intestinal epithelial cell lines (i.e. ICcl2, Mode-K) is supposed to promote a rapid recruitment of immune effector cells in the infected mucosa (35). However, in our co-culture system the number of innate IELs was slightly but not significantly increased in infected organoids compared to the non-infected ones. The composition of the population of innate IELs which infiltrated the organoids was also not modified by the infection. Likewise, no accumulation of immune cells nor modification of the IEL population was observed in the gut of *Rag2^-/-^* mice 1 day after the infection by *C. parvum.* Altogether these data suggested that the impact of immune cells recruitment was insignificant at this very early stage of the infection.

Actually, innate IELs from naive *Rag2^-/-^* mice efficiently blocked the expansion of *C. parvum* in infected organoids attesting that the immune cells which reside within the intestinal epithelium were already armed to fight the parasite. Moreover, we showed that the protection is mainly mediated by CD103^+^ T-cell-like innate IELs which is a dominant subset in the gut epithelium of *Rag2^-/-^* mice (4).

Seeking for the underlying molecular mechanism, we first investigated the contribution of IFN-γ which plays a potent role in cryptosporidiosis. Indeed, several studies have shown that deletion or neutralization of the cytokine increased the parasite burden and aggravate the infection in immunodeficient mice (16–18, 36). In addition, we found that the cytokine was rapidly (i.e. within 24h PI) up-regulated in the whole intestine and in innate IELs isolated from *Rag2^-/-^* mice. However, the amount of IFN-γ was low and was not increased by *C. parvum* infection in co-cultures. Besides, its neutralization with a blocking anti-IFN-γ mAb did not inhibit the antiparasitic effect of innate IELs. Thus, the protection mechanism mediated by innate IELs in co-culture was independent of IFN-γ secretion. This result might be explained by the small number of ILC1s in intestinal organoids as we detected low or no expression of *Ncr1* in co-cultures. The ILC1 subset represents only around 5% of the IELs isolated from *Rag2^-/-^* and thus, the number of ILC1s that colonize organoids may not be sufficient to see their effect. Moreover, osteopontin (*Spp1*) which was shown to promote the homeostasis of intraepithelial ILC1 (6) was not detected in co-cultures (data not shown) and thus their survival could also be impaired. Yet, iCD8α IELs which produce osteopontin were present in co-cultures and *Spp1* expression was significantly increased in the gut of infected *Rag2^-/-^* mice. Further work is then needed to better define the role of osteopontin and ILC1s in cryptosporidiosis.

Nonetheless, ILC1s were not involved in the protective effect observed in co-cultures as their depletion did not affect the growth of *C. parvum* in organoids.

Cytotoxic mechanisms are also involved in the defense against *Cryptosporidium* infections (26–29), we then investigated the role of perforin and granzymes in the protection mediated by innate IELs. Perforin is one of the major effector molecules used by cytotoxic cells to mediate cell lysis since it forms pores in the plasma membrane of target cells that allow the entrance of toxic molecules such as granzymes. Its inhibition impairs cytolytic activity. Herein, we showed that the perforin inhibitor CMA abolishes the protective effect of innate IELs in co-cultures. As shown by others, CMA blocks the acidification of lytic granules and thereby inactivates the cathepsin L required for the maturation of perforin (30, 31). This result indicates that innate IELs can control *C. parvum* growth through a cytotoxic-dependent mechanism. In contrast, we observed a small but non-significant increase of the parasitic burden in co-cultures with innate IELs treated with a granzyme B inhibitor. Thus, Granzyme B has a minor role in this cytotoxic mechanism mediated by innate IELs. Yet, we report a rapid up-regulation of granzyme B expression in the small intestine of infected *Rag2^-/-^* mice that might reflect the activation of cNK cells of the lamina propria (26, 27).

Finally, using aprotinin, a non-selective serine-protease inhibitor, we almost completely restore the expansion of the parasite indicating that other granzymes contribute to the protection mediated by innate IELs in the co-culture. T-cell-like innate IELs are cytotoxic cells and they not only express high amounts of granzyme B but also of granzyme A (4, 5). Besides, the transcriptomic analysis of the iCD8α subset suggest that those cells express additional granzymes such as K and M (5). These proteases could then participate to the cytolytic mechanism.

Interestingly, we also found that infected iEC down regulated the expression of serpinb9b, a natural inhibitor of granzyme M (37), and also that of other serpin b family members. Based on these data, it is tempting to speculate that infected iECs decrease their resistance to granzyme-mediated attacks in order to favor the elimination of the parasite.

## 5 Conclusion

In conclusion, we have developed co-culture model to specifically investigate the role of innate IELs during the very early stages of cryptosporidiosis. This original approach revealed that innate IELs, most likely T-cell-like innate IELs, provide a rapid protection against *C. parvum* infection through a perforin/granzymes-dependent mechanism. Moreover, we showed that the infection modulates functions of iEC that favor the recruitment of effector immune cells and may increase their sensitivity to the cytotoxic attack. Still, further work is needed to detailed the molecular mechanisms involved in these processes.

## Data availability statement

RNAseq data have been deposited in sequence Read Archive (SRA) data with the accession code PRJNA980614, https://www.ncbi.nlm.nih.gov/search/all/?term=PRJNA980614.

## Ethics statement

The animal study was approved by Comité d’éthique en expérimentation animale n°075 APAFIS#30539. The study was conducted in accordance with the local legislation and institutional requirements.

## Author contributions

BM designed and supervised the study. FH, MD, KG, ME, and GC participated in study design and performed research. PZ participated in experiments. BM and FH wrote the manuscript. All authors contributed to the article and approved the submitted version.

## Supplementary material

Supplementary Movie 1 Microinjection of murine organoids with *C. parvum* oocysts in excystation medium stained with fast green dye.
