# Supplementary material for: Cytotoxic innate intraepithelial lymphocytes control early stages of Cryptosporidium infection
Source: Front Immunol. 2023 Sep 6;14:1229406. doi: 10.3389/fimmu.2023.1229406 (PMC10512070; doi:10.3389/fimmu.2023.1229406)
Supplement: Supplementary file 2 [file DataSheet_1.docx]

Supplementary Material

**Cytotoxic innate intraepithelial lymphocytes control early stages of Cryptosporidium infection**

Hariss F^1^, Delbeke M^1^, K Guyot^2^, P Zarnitzky^1^, M Ezzedine^3^, G Certad^2 4^, B Meresse^1^.

^1^ Univ. Lille, Inserm, CHU Lille, U1286 - INFINITE - Institute for Translational Research in Inflammation, F-59000 Lille, France

^2^ Institut Pasteur de Lille, U1019-UMR 9017-CIIL-Centre d'Infection et d'Immunité de Lille, University of Lille, F-59000 Lille, France

^3^ Department of Biology, Faculty of Science, Lebanese University, Beirut, Lebanon

^4^ Délégation à la Recherche Clinique et à l'Innovation, Groupement des Hôpitaux de l'Institut Catholique de Lille, F-59462 Lomme, France

*** Correspondence:**bertrand.meresse@inserm.fr

Keywords: Gut, Innate intraepithelial lymphocytes, Cryptosporidium, Organoids, Cytotoxicity.

# Supplementary Figures and Tables

## Supplementary Figures


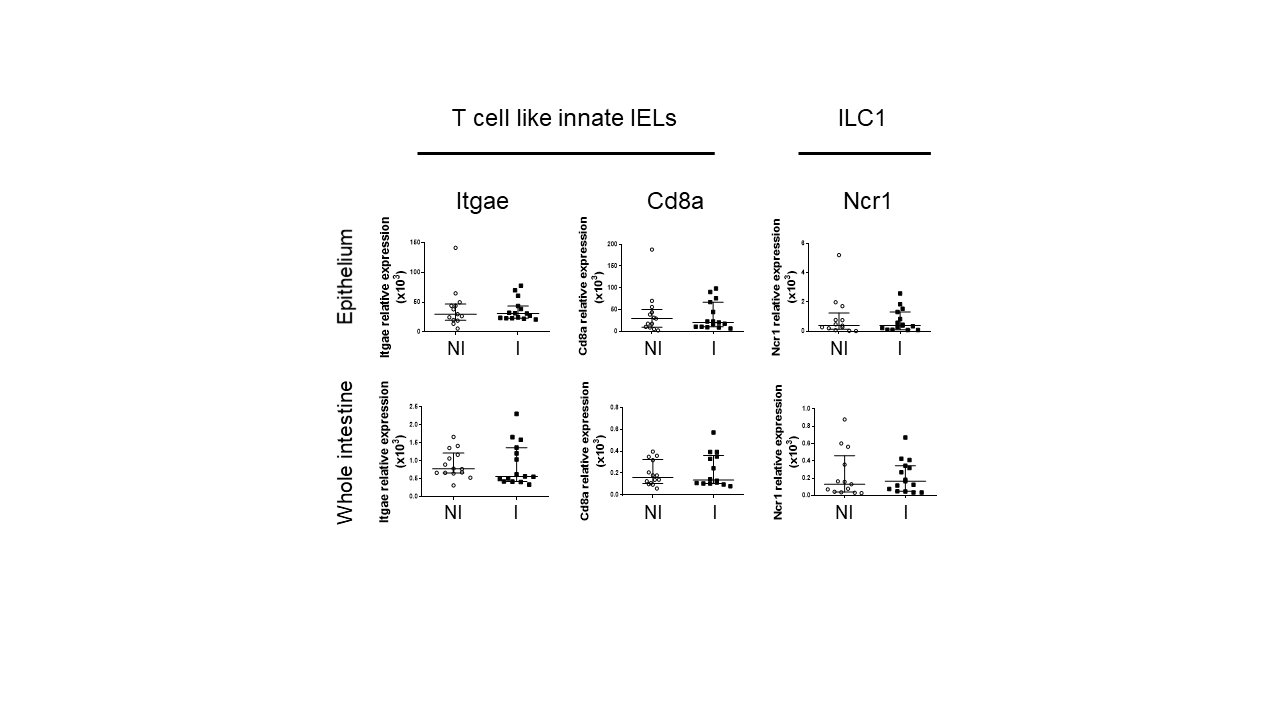


**Supplementary Figure 1.** Quantification of innate T cell like IELs and ILC1 in the gut using their gene signature. Amounts of Itgae and Cd8a mRNA expressed by T cell like IELs and of *Ncr1* mRNA present in ILC1 were quantified in the small intestine and in the epithelium of *Rag2^-/-^* mice infected (I) (n=15) or not (NI) (n=14) with *C parvum* for 24h using qRT-qPCR.


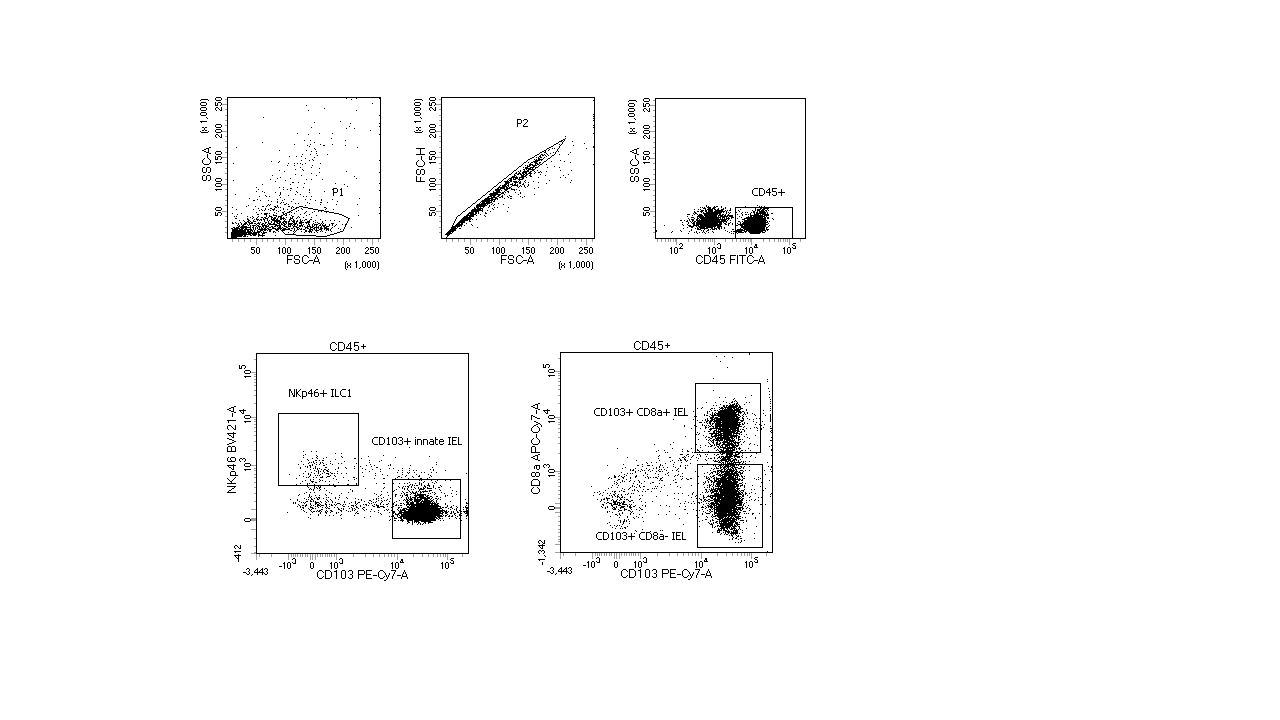


**Supplementary Figure 2.** Ga*ting strategy* for Flow cytometry analysis of innate IELs from *Rag2^-/-^* mice*.* Cells were first selected using forward scatter area (FSC-A)/side scatter area (SSC-A) plot and cell doublets that deviate from the linear correlation between the FSC‐A and the FSC height (FSC‐H) parameters, were excluded. Next CD45^+^ cells were selected and divided based on their expression of NKp46 and CD103 into ILC1(NKp46^+^CD103^-^) and CD103^+^ innate IELs (CD103^+^NKp46^+/-^). CD103^+^ innate IELs were further separated according to their expression of CD8α.


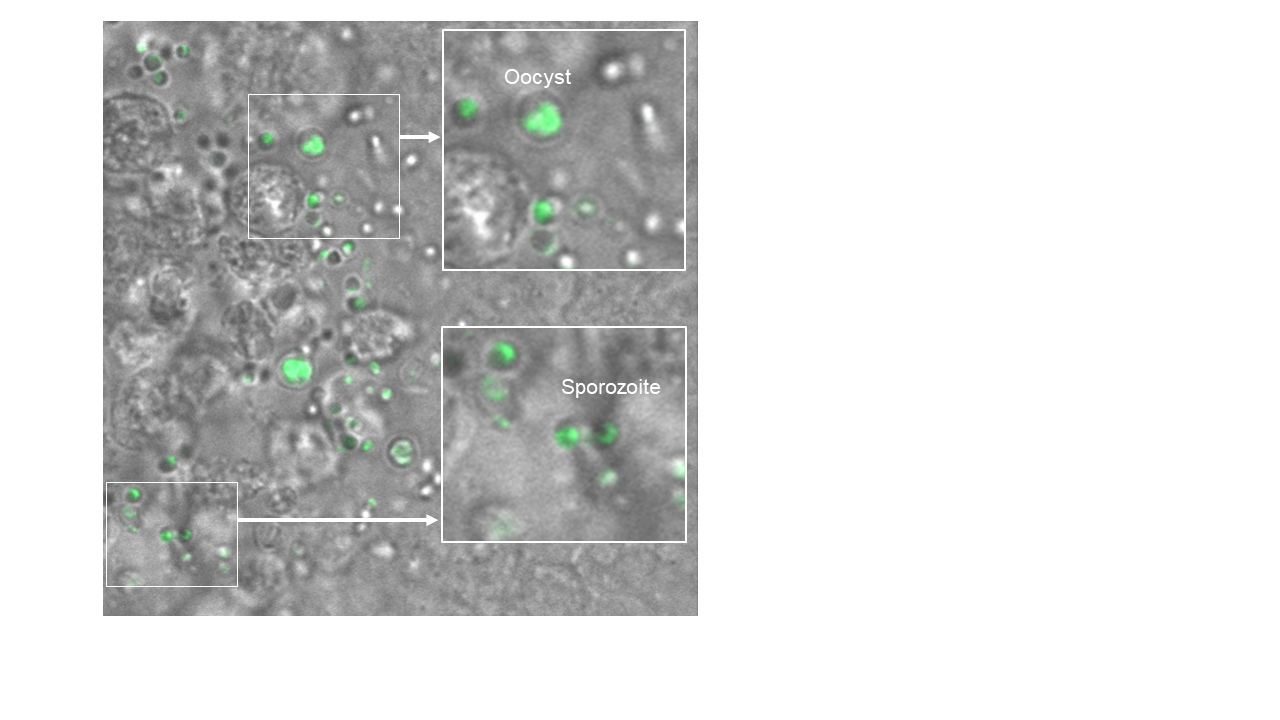


**Supplementary Figure 3.** CSFE-labeled oocysts and sporozoites (green) microinjected inside murine intestinal organoids.


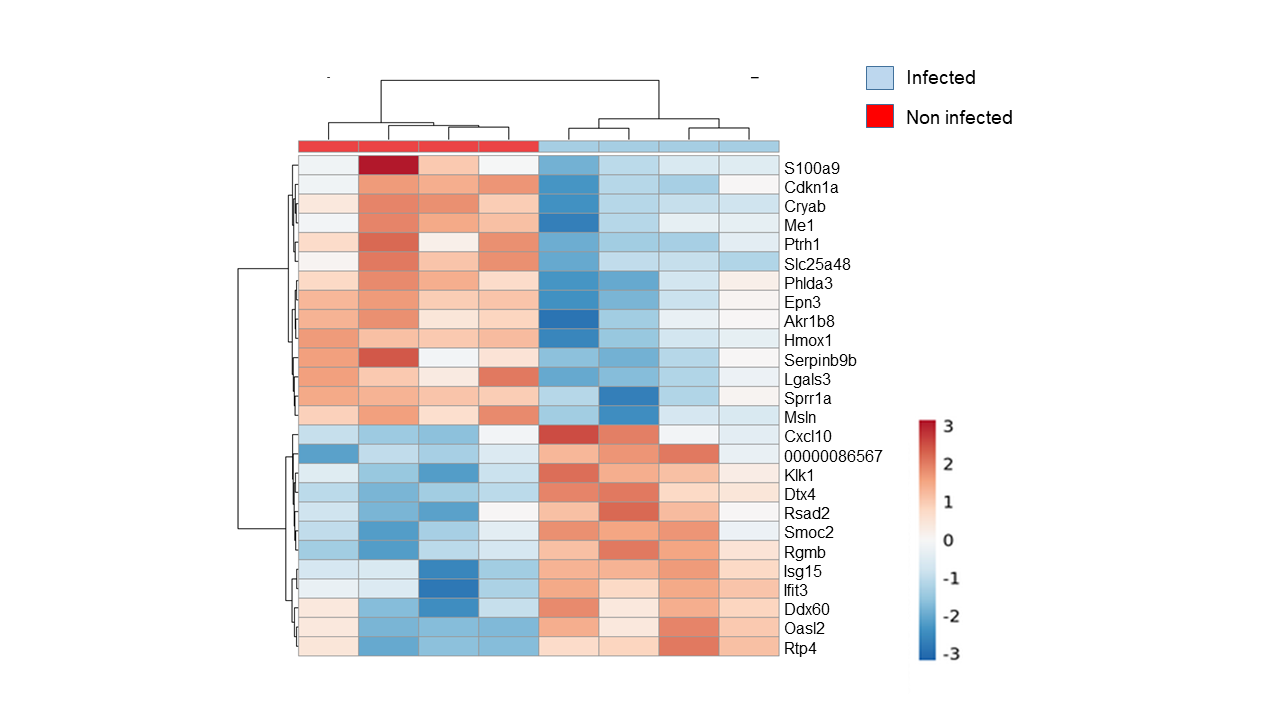


**Supplementary Figure 4.** Heatmap of differentially expressed genes (adjusted p-value ≤ 0.1 and FC ≥ 1.5) between organoids infected with *C.parvum* for 24 h (blue) (n=4) and non-infected organoids (red) (n=4).


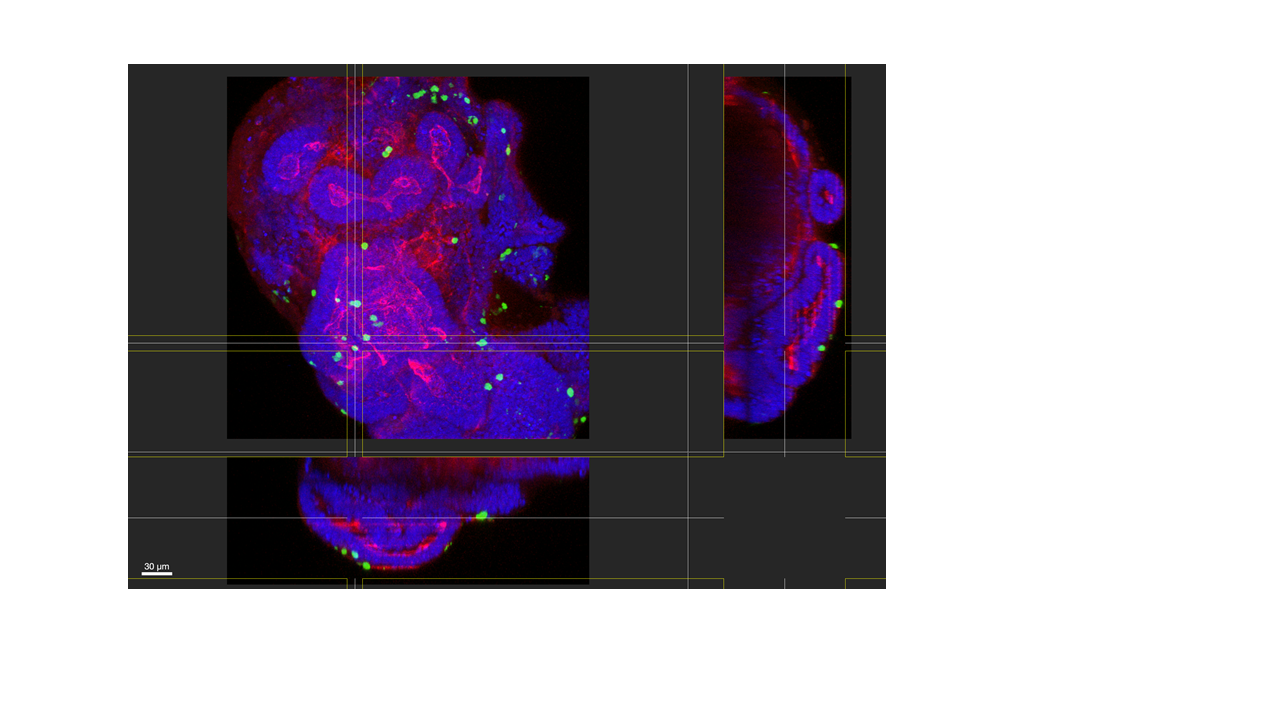


**Supplementary Figure 5.** Three dimensions orthogonal image of murine intestinal organoid co-cultured for 24h with innate IELs. Confocal microscopy imaging with nuclei in blue (DAPI) actin in red (phalloidin) and innate IELs in green (CFSE).


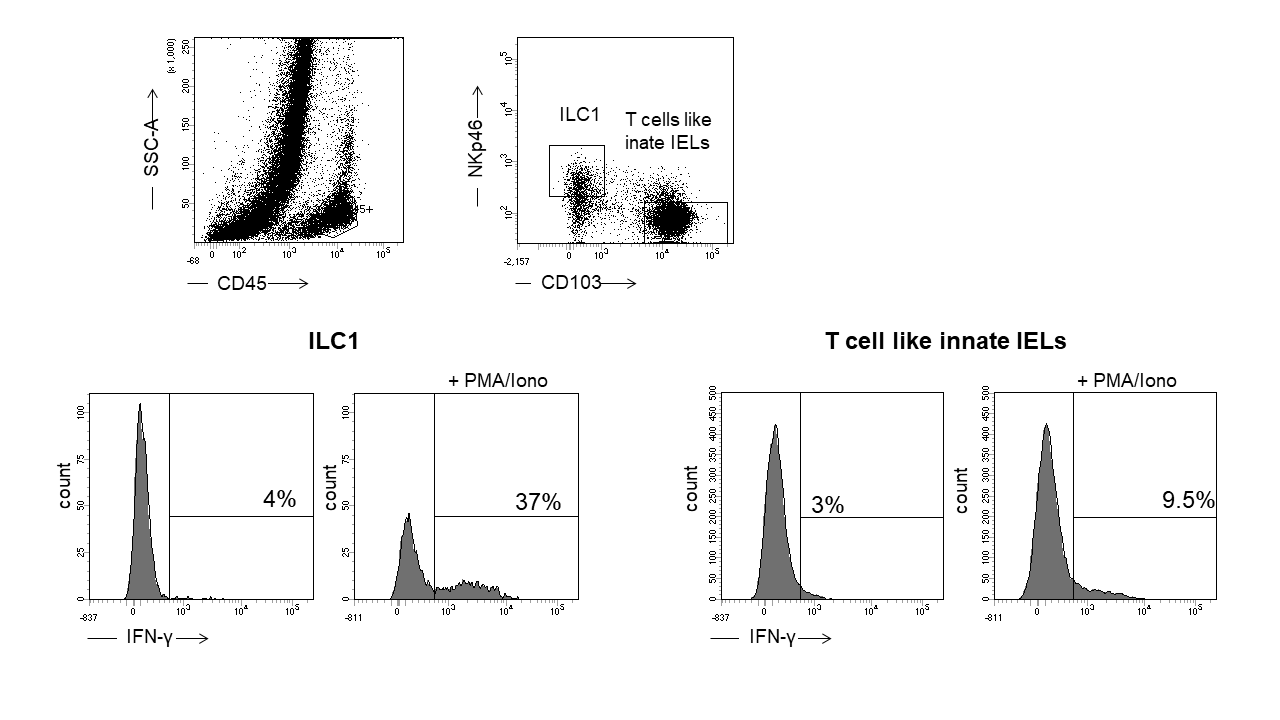


**Supplementary Figure 6.** Production of IFN-γ by subsets of innate IELs. Innate IELs isolated from *Rag2-/-* mice were stimulated in vitro for 5h with PMA-Ionomycine in presence of brefeldin A. Then, cells were stained with a PE-labeled anti-IFN-γ Ab (clone XMG1.2 Sony), a BV421-labeled anti-NKp46 Ab to identify ILC1s and a Pe-Cy7-labeled anti-CD103 Ab to visualize innate T-cell-like IELs.


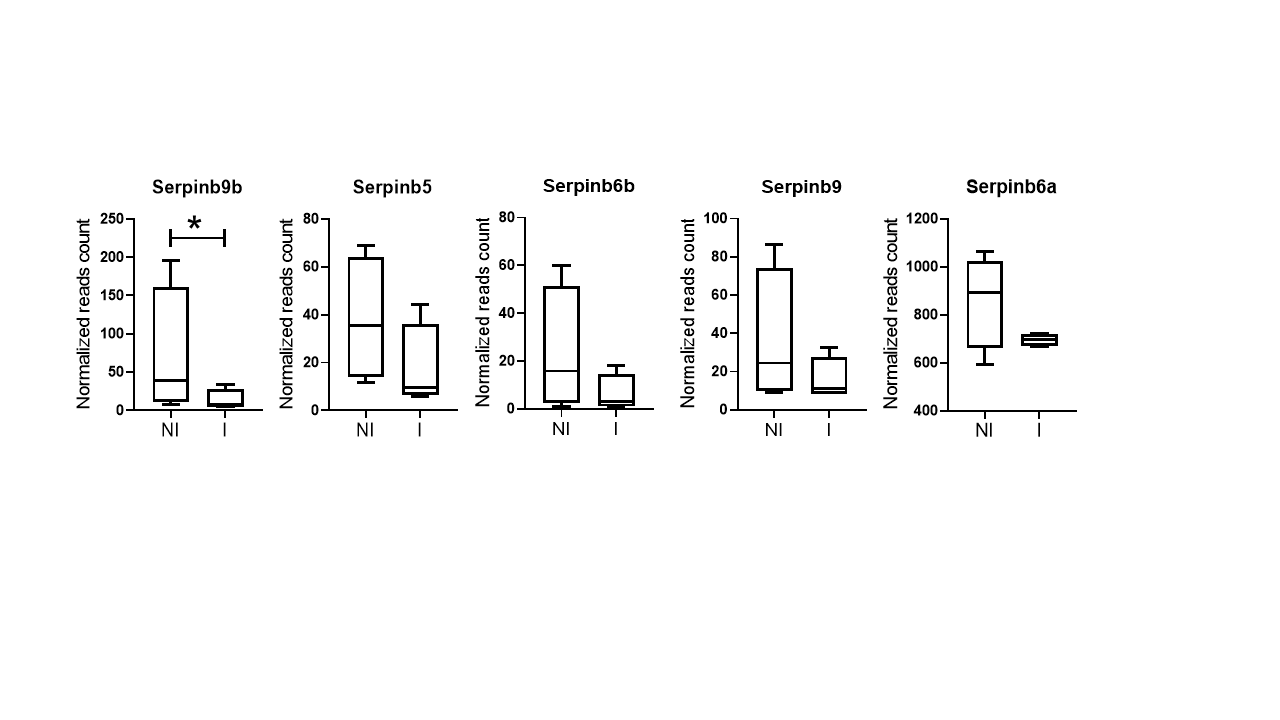


**Supplementary Figure 7.** Expression of serpin family B members using 3’RNA sequencing.

## Supplementary Tables

Tableau S1. GO enrichment analysis based on biological processes (BP)

| **ID** | **Description** | **GeneRatio** | **BgRatio** | **pvalue** | **padjust** | **qvalue** | **GeneID** | **Count** |
| --- | --- | --- | --- | --- | --- | --- | --- | --- |
| GO:0002252 | immune effector process | 10/24 | 432/11202 | 7,9219E-09 | 6,7494E-06 | 5,045E-06 | 15368/16854/20706/  58185/15945/67775/  23962/234311/15959/  100038882 | 10 |
| GO:0051607 | defense response to virus | 7/24 | 165/11202 | 3,7126E-08 | 1,5815E-05 | 1,1822E-05 | 58185/15945/67775/  23962/234311/15959/  100038882 | 7 |
| GO:0009615 | response to virus | 7/24 | 193/11202 | 1,0916E-07 | 3,1002E-05 | 2,3173E-05 | 58185/15945/67775/  23962/234311/15959/  100038882 | 7 |
| GO:0002684 | positive regulation of immune system process | 7/24 | 473/11202 | 4,2354E-05 | 0,00902136 | 0,00674317 | 15368/16854/58185/  15945/12575/234311/  100038882 | 7 |
